# Do the associations of daily steps with mortality and incident cardiovascular disease differ by sedentary time levels? A device-based cohort study

**DOI:** 10.1136/bjsports-2023-107221

**Published:** 2024-03-05

**Authors:** Matthew N Ahmadi, Leandro F M Rezende, Gerson Ferrari, Borja Del Pozo Cruz, I-Min Lee, Emmanuel Stamatakis

**Affiliations:** 1 Mackenzie Wearables Research Hub, Charles Perkins Centre, The University of Sydney, Sydney, New South Wales, Australia; 2 School of Health Sciences, Faculty of Medicine and Health, The University of Sydney, Sydney, New South Wales, Australia; 3 Department of Preventive Medicine, Escola Paulista de Medicina, Universidade Federal de Sao Paulo Escola Paulista de Medicina, Sao Paulo, Brazil; 4 Facultad de Ciencias de la Salud, Universidad Autónoma de Chile, Providencia, Chile; 5 Universidad de Santiago de Chile (USACH), Escuela de Ciencias de la Actividad Física, el Deporte y la Salud, Chile; 6 Department of Physical Education and Sports, Faculty of Education, University of Cádiz, Cádiz, Spain; 7 Biomedical Research and Innovation Institute of Cádiz (INiBICA) Research Unit, University of Cádiz, Cádiz, Spain; 8 Department of Sports Science and Clinical Biomechanics, University of Southern Denmark, Odense, Denmark; 9 Division of Preventive Medicine, Brigham and Women’s Hospital and Harvard Medical School; Department of Epidemiology, Harvard TH Chan School of Public Health, Boston, Massachusetts, USA

**Keywords:** physical activity, sedentary behavior, cardiovascular diseases, death, wearables

## Abstract

**Objectives:**

This study aims to examine the associations of daily step count with all-cause mortality and incident cardiovascular disease (CVD) by sedentary time levels and to determine if the minimal and optimal number of daily steps is modified by high sedentary time.

**Methods:**

Using data from the UK Biobank, this was a prospective dose–response analysis of total daily steps across low (<10.5 hours/day) and high (≥10.5 hours/day) sedentary time (as defined by the inflection point of the adjusted absolute risk of sedentary time with the two outcomes). Mortality and incident CVD was ascertained through 31 October 2021.

**Results:**

Among 72 174 participants (age=61.1±7.8 years), 1633 deaths and 6190 CVD events occurred over 6.9 (±0.8) years of follow-up. Compared with the referent 2200 steps/day (5th percentile), the optimal dose (nadir of the curve) for all-cause mortality ranged between 9000 and 10 500 steps/day for high (HR (95% CI)=0.61 (0.51 to 0.73)) and low (0.69 (0.52 to 0.92)) sedentary time. For incident CVD, there was a subtle gradient of association by sedentary time level with the lowest risk observed at approximately 9700 steps/day for high (0.79 (0.72 to 0.86)) and low (0.71 (0.61 to 0.83)) sedentary time. The minimal dose (steps/day associated with 50% of the optimal dose) of daily steps was between 4000 and 4500 steps/day across sedentary time groups for all-cause mortality and incident CVD.

**Conclusions:**

Any amount of daily steps above the referent 2200 steps/day was associated with lower mortality and incident CVD risk, for low and high sedentary time. Accruing 9000–10 500 steps/day was associated with the lowest mortality risk independent of sedentary time. For a roughly equivalent number of steps/day, the risk of incident CVD was lower for low sedentary time compared with high sedentary time.

**WHAT IS ALREADY KNOWN ON THIS TOPIC**

- Evidence has prompted healthcare professionals to prescribe increasing daily steps as an important intervention to reduce mortality and morbidity.
- High sedentary time is associated with increased risk for mortality and morbidity.
- Evidence is lacking on whether sedentary time modifies modifying effects of sedentary time on the optimal and minimal dose-response of daily steps associated with all-cause mortality and incident cardiovascular disease.

**WHAT THIS STUDY ADDS**

- There was no effect modification by sedentary time levels on the dose–response association of daily steps.
- The lowest mortality risk was observed between 9000 and 10 500 steps/day independent of sedentary time.
- There was about a 10% lower cardiovascular disease risk for an equivalent number of daily steps for low sedentary time compared with high sedentary time.

**HOW THIS STUDY MIGHT AFFECT RESEARCH, PRACTICE OR POLICY**

- These findings provide tangible targets that can be implemented in future daily step count and sedentary time-based interventions.
- Our findings may inform the development of the first steps-based recommendations and future public health physical activity and sedentary time guidelines.

## Introduction

Greater daily steps have established protective effects on health, and its potential benefits have been associated with lower mortality and cardiovascular disease (CVD).^1–3^ Recent studies have found as few as 4000 to 10000 steps are associated with lower mortality and morbidity with potentially continuing risk reductions for higher daily steps.^1 2 4 5^ In contrast, high amounts of sedentary time are associated with higher mortality and morbidity risk.^6–9^ Previous meta-analyses reported a 30%–50% increase in all-cause mortality and CVD from high levels of sedentary time (eg, >10–14 hours/day).^6 7^ Daily steps and sedentary time affect similar risk factors that contribute to the development of CVD and higher mortality risk, such as obesity, blood pressure and cholesterol.^10 11^ However, the current evidence on daily stepping comes from studies that did not consider whether (and to what extent) the association with mortality and incident CVD was modified or attenuated by levels of sedentary time.

Studies examining joint associations and effect modification have reported physical activity may offset or attenuate the higher risk of all-cause mortality^12–15^ and CVD^16–18^ associated with sedentary time. A meta-analysis of self-reported sedentary time and physical activity suggested that 60–75 min/day of moderate-to-vigorous physical activity (MVPA) lowered the detrimental associations of high sedentary time,^19^ while data from the 45 and Up Study showed high sedentary time was only associated with higher mortality risk in those not attaining the minimum threshold of current recommendations (at least 150 MVPA min/week).^18^ A harmonised meta-analysis of hip worn accelerometerdevices suggested that 30–40 min/day of MVPA attenuated the all-cause mortality risk attributed to sedentary time.^20^ Collectively, this body of evidence estimated time in intensity-specific physical activity needed to offset or substantially attenuate high levels of predominantly self-reported sedentary time. For many individuals, it may be challenging to recall time or estimate intensity to determine whether they are sufficiently active in relation to minute-based and intensity-based targets. Stepping-based information may provide a more tangible physical activity prescription that is easier to act on.

No study to date has examined if high sedentary time modifies the dose-response of daily steps with all-cause mortality and incident CVD. Such information can be used to advise the general public, inform guidelines and improve clinical intervention targets. Importantly, with the proliferation of wearable devices, steps-based and sedentary time-based health information could be streamlined through consumer wearables, making it easier to self-monitor levels, set goals and potentially improve physical activity promotion.^21 22^

We aimed to determine if sedentary time modified the optimal and minimal daily steps associated with all-cause mortality and incident CVD risk. We pursued these aims by examining the detailed dose response of daily steps across high and low sedentary time levels in a large cohort of UK adults using wrist-worn accelerometers.

## Methods

### Study participants

Participants were included from the UK Biobank Study, a prospective cohort of 502 629 participants between 40 and 69 years. All participants were enrolled between 2006 and 2010 and provided informed written consent. Participants completed physical examinations by trained staff and touchscreen questionnaires. We excluded participants with diagnosed CVD or cancer (ascertained through self-report, hospital admission and cancer registry records) prior to accelerometry measurement, missing covariate data or an event within the first 12 months from the accelerometry measurement (online supplemental figure 1).

10.1136/bjsports-2023-107221.supp1 Supplementary data

### Steps and sedentary time assessment

From 2013 to 2015, 103 684 participants were mailed and wore an Axivity AX3 accelerometer (Newcastle upon Tyne, UK) on their dominant wrist for 24 hours/day for 7 days to measure physical activity. Prior to being mailed, the AX3 accelerometers were initialised to collect data with a sampling frequency of 100 Hz and a dynamic range between±8 g. Participants returned the devices by mail and the data were calibrated and non-wear periods were identified according to standard procedures.^23 24^ Monitoring days were considered valid if wear time was greater than 16 hours. In this study, participants were required to have at least three valid monitoring days, with at least one of those days being a weekend day, and have worn the monitor during sleep periods. Physical activity type was classified with a validated accelerometer-based activity machine learning scheme covering sedentary behaviour, small utilitarian movements, walking and running,^25 26^ consistent with previously published studies.^3 5 27^ We calculated steps during periods of ambulation using a tuned signal peak detection method^28 29^ used in previous studies^3 5^ and in validation studies shown to have a step detection accuracy of 89%^29^ and a total steps mean absolute percent error of 10%^28^ and a mean bias of 9%.^30^ A complete description of the step detection and internal validation is provided in online supplemental text 1. Primary exposures were daily time spent sedentary (based on absolute risk curves categorised as: low <10.5 hours/day and high ≥10.5 hours/day), and daily step counts.

### Mortality and cardiovascular disease ascertainment

Participants were followed up to 30 September 2021 (England and Wales) or 31 October 2021 (Scotland), with deaths obtained through linkage with the NHS Digital of England and Wales or the NHS Central Register and National Records of Scotland. Inpatient hospitalisation data (England: 30 September 2021; Scotland: 31 July 2021; Wales 28 February 2018) were provided by either the Hospital Episode Statistics for England, the Patient Episode Database for Wales or the Scottish Morbidity Record for Scotland. CVD was defined as diseases of the circulatory system, excluding hypertension, diseases of arteries and lymphatic system. Online supplemental table 1 describes in detail CVD ascertainment methods.

### Covariates

Our selection of covariates was based on previous daily stepping and sedentary time literature (online supplemental figure 2) and included age, sex, ethnicity, education, smoking status, alcohol consumption, fruit and vegetable consumption (servings per day), parental history of CVD and cancer, medication use (cholesterol, insulin and hypertension) and accelerometer-measured sleep time (hours/day). In sensitivity analyses, we included clinical factors that may be potential mediators: waist circumference, glycated haemoglobin A1C, high-density and low-density lipoprotein, diastolic and systolic blood pressure, and triglycerides. Complete covariate definitions are provided in online supplemental table 2.

### Analyses

We calculated the adjusted dose–response absolute risk for all-cause mortality and incident CVD per 10 000 person-years, and crude risk percent (categorical). We used Cox proportional hazards regression models to estimate HR with 95% CIs for all-cause mortality. Fine-Gray subdistribution method was used for incident CVD analyses with non-cardiovascular deaths treated as a competing risk. In both sets of analyses, we used restricted cubic splines with knots at the 10th, 50th and 90th percentile to model the dose–response associations. No violations in any of the assumptions of Cox proportional hazard model were observed. Specifically, we checked for assumptions of Cox proportional hazard model including Schoenfeld residual, independence of survival times for individuals, linearity of covariates, continuous survival time, multicollinearity and independence of censoring date, and no violations were observed. Effect modification was tested by fitting an interaction term between sedentary time and daily steps. We examined the dose response for the optimal (nadir of the curve) and minimal (defined as 50% of the optimal dose^5 27^; ((1−opimal dose HR)/2) number of steps for high and low sedentary time. In all analyses, we set the reference data point to be the 5th percentile of daily steps among all participants (eg, 2200 steps).

We calculated E-values for the optimal and minimal daily steps to estimate the plausibility of bias from unmeasured confounding.^31^ To assess the robustness of our findings, we performed additional joint association analyses with 2200 daily steps (congruent with stratified analysis) and high sedentary time as the reference. In sensitivity analyses, we adjusted for clinical factors (see covariate section above) that could be considered mediators of the association between the exposures and outcomes. We further performed an analysis with alternate sedentary time groupings with the highest quartile (≥11.5 hours/day) categorised high sedentary time and the lowest three quartiles as low sedentary time. We also conducted sensitivity analyses to examine reverse causation bias by excluding underweight participants (body mass index <18.5 kg/m^2^), participants reporting self-rated fair or poor health, or participants with an event within the first 2 years of follow-up.^32 33^ We also assessed incident CVD risk using cause-specific analysis to provide estimates of direct effects.^34 35^ In addition, we assessed age subgroup differences for participants <60 years old and ≥60 years old using an interaction term for age in our incident CVD analysis.

We performed all analyses using R statistical software. We reported this study as per the Strengthening the Reporting of Observational Studies in Epidemiology guideline and the Checklist for statistical Assessment of Medical Papers.^36^

### Patient and public involvement

No patients or members of the public were involved in the planning, design, data collection, analysis or interpretation of results for this study.

### Equity, diversity and inclusion statement

Our study sample representative of all participants who participated in the UK Biobank study with valid accelerometer data, reflecting the demographic, geographical and socioeconomic diversity of the participants.

## Results

Our analytical sample for mortality included 72 174 participants (average age (SD)= 61.1 (7.8) years; 57.9% female) followed up for an average of 6.9±0.8 years with 1633 deaths. Our incident CVD analysis sample included 71 441 participants with 6190 events. Median (IQR) total steps and sedentary time were 6222 (4102–9225) steps/day and 10.6 (9.7–11.6) hours/day, respectively. Participants wore the accelerometers for an average of 22.8 hours/day. Participant characteristics by sedentary time are provided in table 1. Participants classified as having high sedentary time (53.8% of the total sample) were more likely to be current smokers, use cholesterol and hypertension medication, and have higher central adiposity (waist circumference) compared with their low sedentary time counterparts. Within the high and low sedentary time levels, median daily steps were 4829 (3329, 6834) and 8362 (5883, 11 792), respectively.

**Table 1:** Participant characteristics by sedentary time

	Sedentary time (hours/day)		
	Low (<10.5)	High (≥10.5)	Overall
Sample	33 338	38 836	72 174
Follow-up, years	7.0 (0.8)	6.9 (0.8)	6.9 (0.8)
Age, years	60.0 (7.8)	62.1 (7.7)	61.1 (7.8)
Steps, (median (IQR))	8362.2 (5883.0–11 791.9)	4829.8 (3329.5–6834.1)	6222.5 (4102.1–9225.4)
Sedentary time, (median (IQR))	9.6 (8.9–10.1)	11.5 (11.0–12.2)	10.6 (9.7–11.6)
Sleep, hours, (median (IQR))	7.8 (6.9–8.6)	7.1 (6.0–7.9)	7.4 (6.4–8.2)
Male, %	12 780 (38.3)	17 570 (45.2)	30 350 (42.1)
Smoking history, %			
Never	19 880 (59.6)	22 290 (57.4)	42 170 (58.4)
Previous	11 347 (34.0)	13 700 (35.3)	25 047 (34.7)
Current	2111 (6.3)	2846 (7.3)	4957 (6.9)
Alcohol consumption, %			
Never	897 (2.7)	1137 (2.9)	2034 (2.8)
Previous	776 (2.3)	1116 (2.9)	1892 (2.6)
Occasional	6408 (19.2)	8287 (21.3)	14 695 (20.4)
Within guidelines	12 576 (37.7)	14 163 (36.5)	26 739 (37.0)
Double guidelines	8113 (24.3)	8804 (22.7)	16 917 (23.4)
More than double guidelines	4568 (13.7)	5329 (13.7)	9897 (13.7)
Education, %			
College/University	14 485 (43.4)	17 715 (45.6)	32 200 (44.6)
A/AS	4504 (13.5)	5171 (13.3)	9675 (13.4)
O levels	6978 (20.9)	7623 (19.6)	14 601 (20.2)
CSE	1624 (4.9)	1322 (3.4)	2946 (4.1)
NVQ/HND/HNC	1708 (5.1)	1988 (5.1)	3696 (5.1)
Other	4039 (12.1)	5017 (12.9)	9056 (12.5)
Diet, servings/day	8.3 (4.5)	8.0 (4.4)	8.1 (4.4)
Parental history of CVD	17 572 (52.7)	21 260 (54.7)	38 832 (53.8)
Parental history of cancer	8200 (24.6)	9820 (25.3)	18 020 (25.0)
Ethnicity, %			
Asian	341 (1.0)	473 (1.2)	814 (1.1)
Black	232 (0.7)	390 (1.0)	622 (0.9)
Mixed	176 (0.5)	237 (0.6)	413 (0.6)
Other	242 (0.7)	348 (0.9)	590 (0.8)
White	32 347 (97.0)	37 388 (96.3)	69 735 (96.6)
Medication use, %			
Cholesterol	2859 (8.6)	5150 (13.3)	8009 (11.1)
Blood pressure	3549 (10.6)	6431 (16.6)	9980 (13.8)
Insulin	147 (0.4)	272 (0.7)	419 (0.6)
Biomarkers			
Glycated haemoglobin	34.7 (4.6)	35.5 (5.8)	35.1 (5.3)
High density lipoprotein	1.5 (0.4)	1.5 (0.4)	1.5 (0.4)
Low density lipoprotein	3.6 (0.8)	3.6 (0.8)	3.6 (0.8)
Triglycerides	1.6 (0.9)	1.7 (1.0)	1.6 (1.0)
Self-rated health, %			
Excellent	8554 (25.7)	8333 (21.5)	16 887 (23.4)
Good	20 393 (61.2)	23 481 (60.5)	43 874 (60.8)
Fair	3887 (11.7)	6112 (15.7)	9999 (13.9)
Poor	466 (1.4)	852 (2.2)	1318 (1.8)
Blood pressure, mm Hg			
Systolic	136.5 (19.0)	139.3 (19.2)	138.0 (19.1)
Diastolic	80.8 (10.5)	82.3 (10.6)	81.6 (10.5)
Body mass index, kg/m^2^	25.7 (4.1)	27.2 (4.6)	26.5 (4.5)
Waist circumference, cm			
Male	92.8 (9.8)	96.4 (11.0)	94.9 (10.6)
Female	80.2 (10.7)	84.4 (12.1)	82.4 (11.6)

Values represent mean (SD) unless stated otherwise.

CVD, cardiovascular disease; HNC, higher national certificate; HND, higher National diploma; NVQ, national vocational qualification.

### Absolute risks

**Figure 1:**
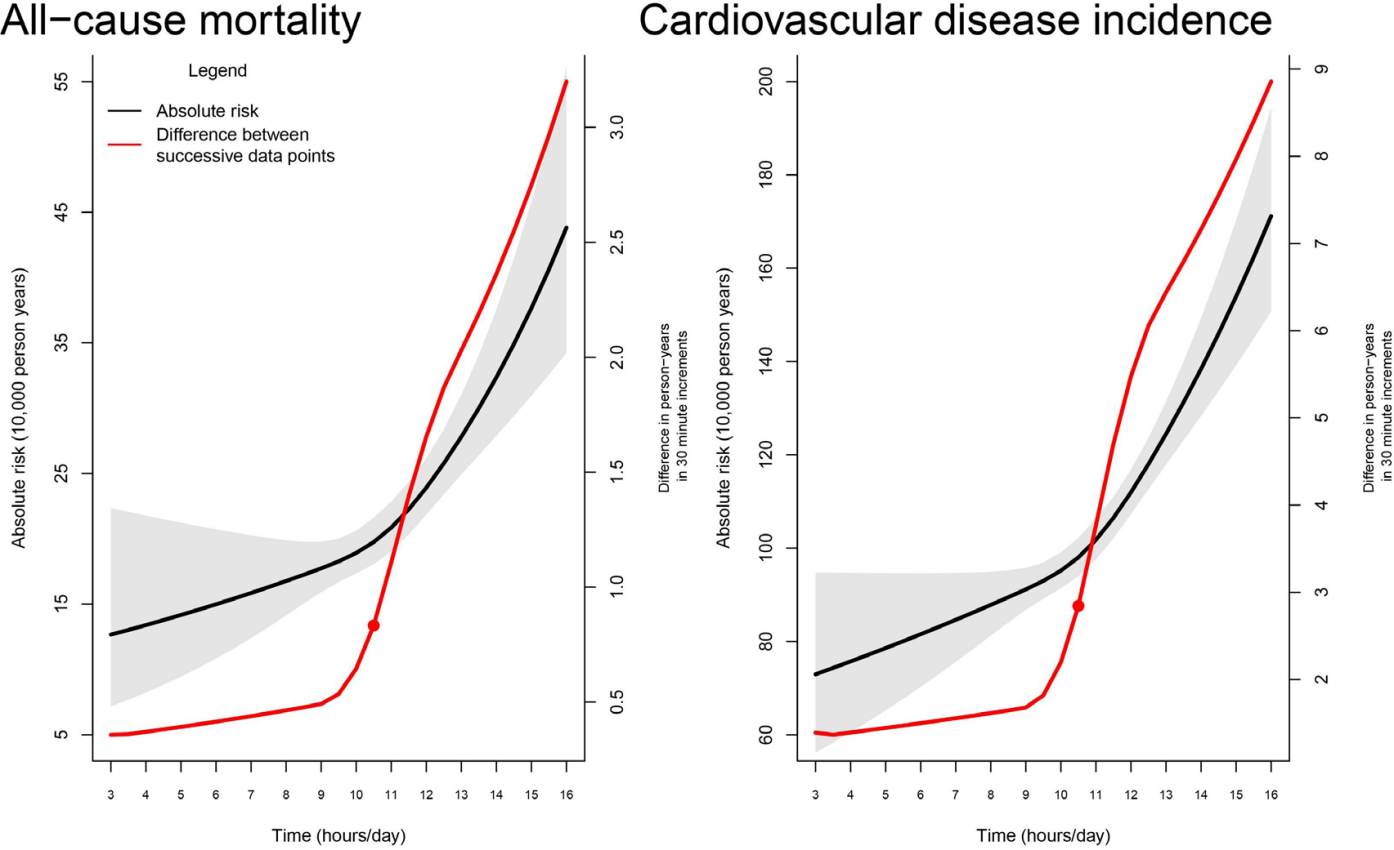
Age-adjusted and sex-adjusted sedentary time dose–response absolute risk for all-cause mortality, and cardiovascular disease incidence. Shaded area represents 95% CI. Red circle indicates delineation between high and low sedentary time

The sex-adjusted and age-adjusted sedentary time dose–response absolute risk for all-cause mortality and incident CVD is shown in figure 1. We used the dose–response results to categorise participants as having high or low sedentary, reflective of when risk became pronounced. Using the difference between adjacent absolute risk estimates in 30 min increments, we found risk became more pronounced for both all-cause mortality and incident CVD at 10.5 hours/day of sedentary time.

**Figure 2:**
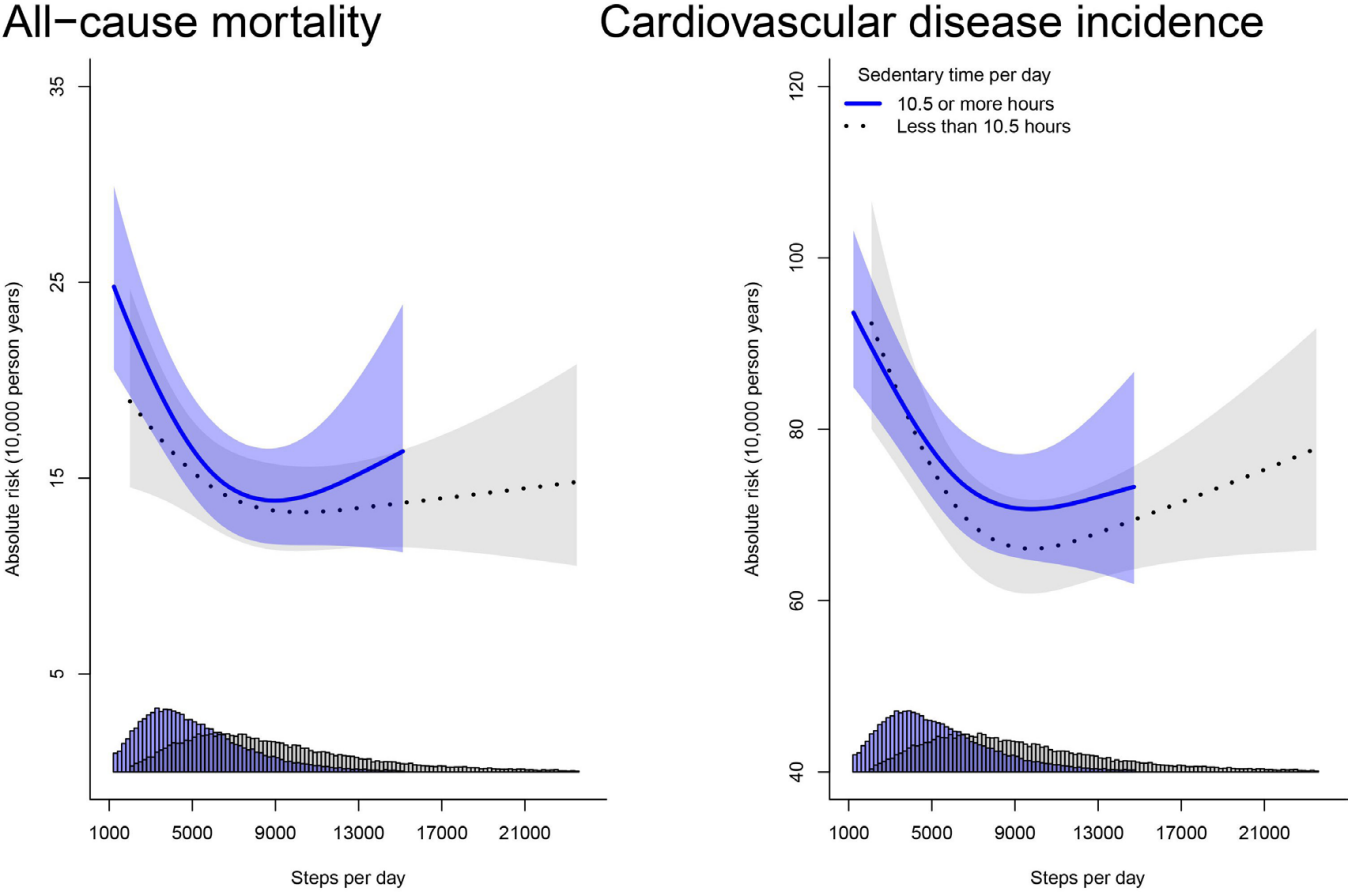
Adjusted absolute risk for all-cause mortality and cardiovascular disease incidence. Adjusted for age, sex, ethnicity, education, smoking status, alcohol consumption, diet, parental history of CVD and cancer, medication use (cholesterol, insulin and hypertension), sleep duration. Shaded area represents 95% CI. CVD, cardiovascular disease.

Online supplemental table 3 and figure 2 present the crude risk and the multivariable-adjusted dose response of all-cause mortality and incident CVD associated steps/day by sedentary time level, respectively. Within the high sedentary time level (≥10.5 hours/day), accumulating <4000 steps/day (tertile 1) was associated with a crude mortality risk of 5.41% (95% CI 5.32% to 5.50%), whereas accumulating >8000 steps/day (tertile 3) was associated with a 3.05% (95% CI 2.96% to 3.13%) crude risk. The corresponding crude risk for participants within the low sedentary time level (<10.5 hours/day) was 3.74% (95% CI 3.62% to 3.86%) and 2.27% (95% CI 2.24% to 2.30%).

### All-cause mortality

**Figure 3:**
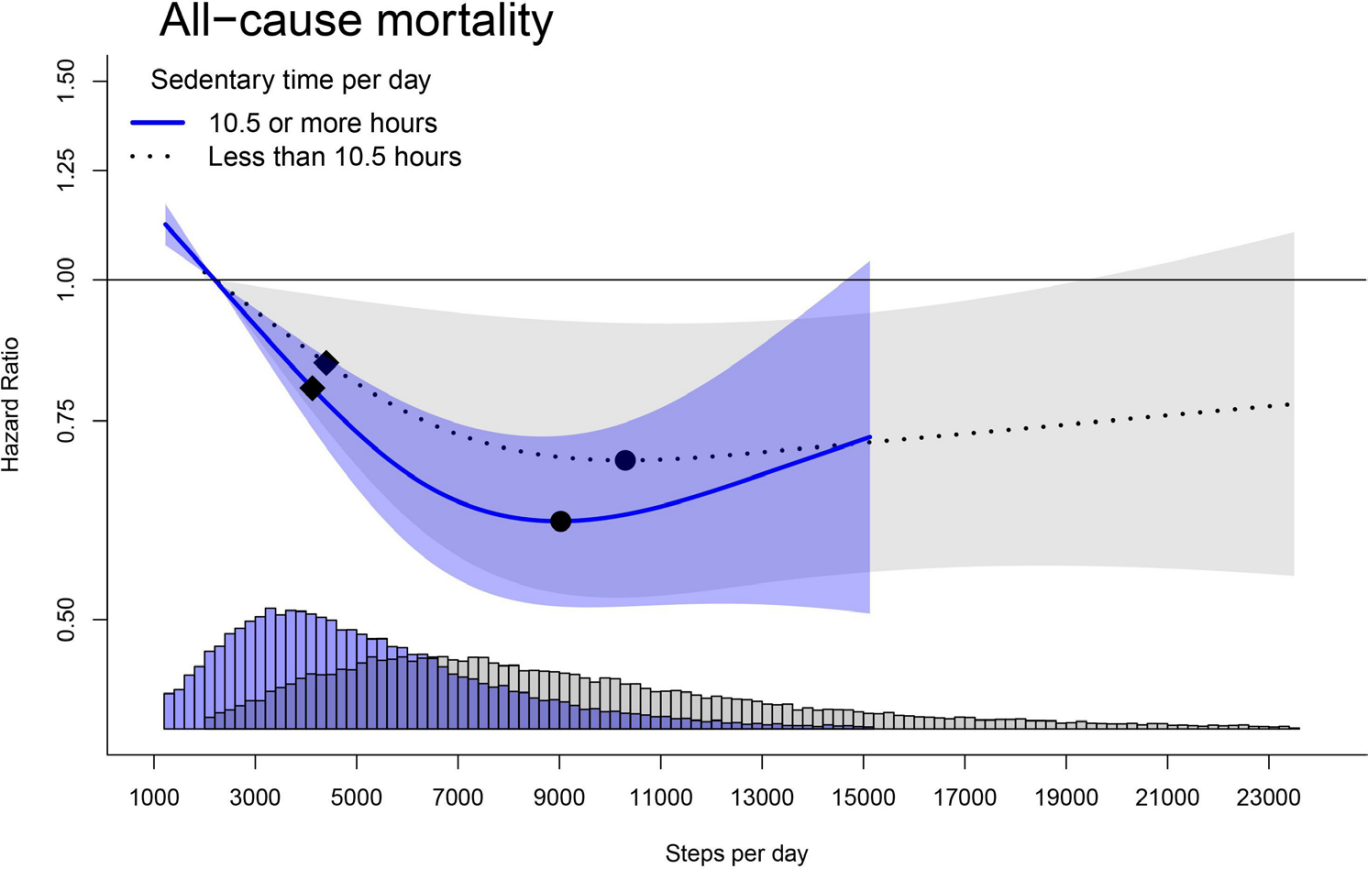
Stratified dose–response association of all-cause mortality and steps by sedentary time. Adjusted for age, sex, ethnicity, education, smoking status, alcohol consumption, diet, parental history of CVD and cancer, medication use (cholesterol, insulin and hypertension) and sleep duration. Shaded area represents 95% CI. Square=minimum dose (ED50); circle=optimum dose (nadir of curve). CVD, cardiovascular disease.

Among participants with high sedentary time, we observed the nadir of the curve at 9000 steps/day corresponding to an HR (95%CI) of 0.61 (0.51 to 0.73), compared with the referent 2200 steps/day (figure 3; effect modification p=0.756). The minimal dose was at 4100 steps/day with an HR of 0.80 (0.74 to 0.87). Among participants with low sedentary time, we observed an attenuation in the magnitude of the steps/day dose–response association with the nadir of the curve at 10 300 steps/day (0.69 (0.52 to 0.92)). We observed the minimal dose at 4400 steps/day with a corresponding HR of 0.84 (0.74 to 0.97). In our joint dose–response analysis (online supplemental figure 3), we observed consistent nadir and minimal dose values between the two sedentary time levels. The mortality risk was similar (eg, HR difference ≤0.03 units) between high and low sedentary time levels at 6000 steps/day and continued to be similar up to 9500 steps/day.

### Incident cardiovascular disease

**Figure 4:**
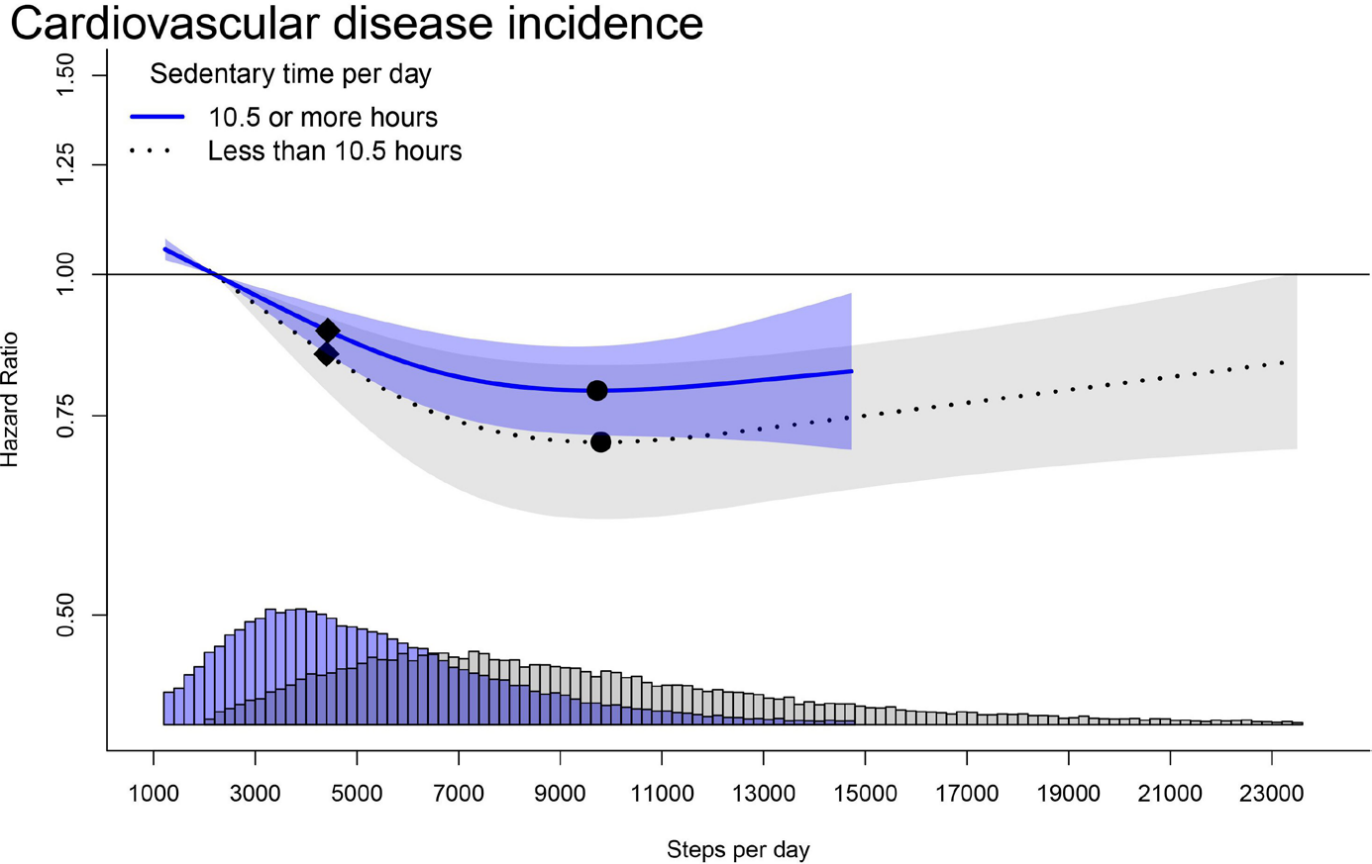
Stratified dose–response association of cardiovascular disease incidence and steps by sedentary time. Adjusted for age, sex, ethnicity, education, smoking status, alcohol consumption, diet, parental history of CVD and cancer, medication use (cholesterol, insulin and hypertension) and sleep duration. Shaded area represents 95% CI. Square=minimum dose (ED50); circle=optimum dose (nadir of curve). CVD, cardiovascular disease.

In the dose–response association between steps/day and incident CVD, we observed lower risk for the low sedentary time group, for an equivalent steps/day, compared with the high sedentary time group (figure 4; effect modification p=0.725). The HR differences between the two groups increased up to the nadir of both curves. The minimal dose was at 4300 steps/day for both high and low sedentary time with corresponding HR’s of 0.90 (95% CI 0.86 to 0.94) and 0.86 (95% CI 0.80 to 0.92). For high sedentary time, the optimal dose (nadir) was at 9700 steps/day with an HR of 0.79 (95% CI 0.72 to 0.86). In comparison, among participants with low sedentary time, we observed a similar optimal dose (9800 steps/day), with a lower corresponding HR of 0.71 (95% CI 0.61 to 0.83). In our joint dose–response analysis (online supplemental figure 4), the lower risk for an equivalent steps/day for low sedentary time compared with high sedentary time was consistent with our main analysis when steps/day exceeded 3700.

### Additional and sensitivity analyses

When adjusting for waist circumference, glycated haemoglobin A1C, high-density and low-density lipoprotein, blood pressure and triglycerides, the association patterns remained consistent, although the magnitude was attenuated for high sedentary time and all-cause mortality (online supplemental figure 5). Exclusion of participants who had fair or poor self-rated health, were underweight or had an event within the first 2 years of follow-up showed generally consistent associations as our main analysis (online supplemental figure 6). For example, in the high sedentary time group 8700 steps/day was associated with the lowest all-cause mortality risk, and among the low sedentary time group the lowest risk was observed at 11 000 steps/day. Alternate sedentary time grouping with the highest quartile (≥11.5 hours/day; high sedentary time) and lowest three quartiles (low sedentary time) showed a consistent dose–response association pattern for incident CVD, and a higher magnitude of association for low sedentary time with all-cause mortality (online supplemental figures 7 and 8). Our E-values suggest a moderate degree of unmeasured confounding would be required to reduce our observed associations for mortality and incident CVD. For example, the minimal steps/day dose E-value ranged from 1.67 (1.21) to 1.81 (1.56) for all-cause mortality and 1.46 (1.32) to 1.60 (1.39) for incident CVD (online supplemental table 4). Cause-specific hazard analysis for incident CVD risk was similar to Fine-Gray subdistribution hazard analysis. For example, in cause-specific analysis the optimal dose was approximately 9600 steps/day for high sedentary time, and 9800 steps/day for low sedentary time (online supplemental figure 9). Subgroup analysis by age showed no association between steps and incident CVD risk among young participants (<60 years old) with low sedentary time. However, among young participants with high sedentary time, there was an inverse association with no upper limit for daily steps and lower incident CVD risk. Among older participants (≥60 years old), we observed lower risk for both low and high sedentary time, with lowest risk observed among older participants with low sedentary time (eg.<10.5 hours/day) at an equal number of daily steps. For older adults with high sedentary time, the lowest risk was observed at approximately 8500 steps/day (online supplemental figure 10; effect modification p=0.153).

## Discussion

Our study adds new evidence to the literature by examining the dose–response association of daily steps with mortality and incident CVD risk in high and low sedentary time groups. For all-cause mortality, the optimal dose occurred between 9000 and 10 500 steps/day across sedentary time groups. Within the high sedentary time group we observed lower risk compared with the low sedentary time group at an equivalent number of daily steps. We found a lower incident CVD risk for an equivalent number of daily steps within the low sedentary time group compared with the high sedentary time group. There was consistency in the optimal and minimal steps/day association with incident CVD risk between the two groups at just under 10 000 steps/day and 4500 steps/day, respectively.

### All-cause mortality

Previous prospective studies examining daily steps did not consider the potential effects of differing sedentary time levels on the association with health risks.^1 37^ Given the established dynamic between physical activity and sedentary time,^17 18^ such an exclusion may lead to overestimation of effect estimates and underestimation of the minimal and optimal steps/day dose response. Studies and meta-analyses assessing daily steps and all-cause mortality, which did not consider sedentary time, showed a curvilinear dose response that suggested between 6000 and 10 000 steps/day was associated with lower all-cause mortality.^1–4 38^ Our analyses expands on previous research and examines the influence of sedentary time on the daily stepping dose-response association. Between 6000 and 10 500 steps/day, we found mortality risk was about 10% lower for an equivalent number of steps in the high sedentary time group compared with the low sedentary time group. Our findings emphasise the importance of increasing daily steps particularly among adults who are highly sedentary. In the high sedentary time group, the stronger association could be attributable to the more pronounced impact of daily step accumulation in individuals who are at a higher risk of mortality from the adverse effects of sedentary time. Among the high sedentary time group, being sufficiently active through daily step accumulation may ameliorate downstream effects of sedentary time, lowering the risk of developing comorbidities and subsequently leading to lower mortality risk.^39 40^ If confirmed in future studies, our dose–response findings may help to improve health messaging and goal setting for the most at-risk individuals in the population.

### Incident cardiovascular disease

We observed lower incident CVD risk for an equivalent number of daily steps for low sedentary time compared with high sedentary time, although with overlapping 95% CIs. This graded association pattern may be due to the separate contributions of sedentary time and daily steps (eg, physical activity) to cardiovascular health, leading to an additive effect on CVD risk. Our cause-specific hazards dose–response analysis, which provides a direct effect estimation, was comparable to our Fine-Gray subdistribution hazards analysis that provides an estimation of the direct and indirect effect estimation.^34^ Studies have demonstrated prolonged sedentary time contributes to increased inflammation, oxidative stress and induces adverse effects on cardiovascular autonomic nervous system function.^41–43^ In contrast, higher daily steps can lead to cardioprotective adaptations.^44–46^ We did not find evidence that daily steps could compensate for excess sitting time. This contrasts prior studies that have found MVPA can lower the risk of high sedentary time to be comparable to low sedentary time.^17 18 47^ The majority of daily steps occur at a light intensity^1 3^ and may explain in part the disparate findings between our study and MVPA intensity focused studies. Taken together, this suggests an important role of physical activity intensity to reduce the risks of sedentary time for CVD prevention.

Among the high sedentary time group, we found a 10%–21% lower CVD risk when daily step accumulation was between 4000 and 10 000 steps/day. The magnitude in the dose–response association we observed for steps/day with CVD risk was attenuated in comparison with two prior meta-analyses that did not account for differing levels of sedentary time.^37 48^ In addition, a prior meta-analysis^37^ of eight cohorts found there was no association between daily steps and lower incident CVD risk among participants <60 years old. Our results extend on this prior finding to provide nuanced information on the influence of sedentary time. Indeed, among adults <60 years old with low sedentary time (<10.5 hours/day), we did not find an association between daily steps and incident CVD risk. However, among adults <60 years old with high sedentary time, we observed an inverse linear association. This finding further highlights the potential health-benefits of increasing daily steps to mitigate CVD risk among highly sedentary adults. The absence of an association among adults <60 years old with low sedentary time could be due to the latency period for CVD to progress towards clinical endpoints of hospitalisations and death compared with their counterparts who have high sedentary time and are at a higher risk of cardiovascular events earlier in adulthood. Collectively, our results underscore the importance for a combination of decreasing sedentary time and increasing daily steps to improve cardiovascular health.

### Implications

Our findings provide new insights regarding the dose response of daily steps, sedentary time, mortality and CVD risk. Overall, between 9000 and 10 500 steps/day was the optimum dose to lower mortality and CVD risk across sedentary time groups. Our prospective results provide relevant findings that can be used to augment public health messaging and inform the first generation of stepping-based and device-based physical activity and sedentary guidelines. Daily stepping targets are a simple metric clinicians and allied health providers can use to monitor and promote physical activity to their patients. Collectively, our findings may have important implications to help improve the efficacy of future trials and the precision of intervention treatments among individuals with varying physical activity and sedentary time levels.

Our results indicate sedentary time did not significantly modify the dose-response association of daily steps. We also found the amount of physical activity (eg, steps/day) needed to lower the risk of mortality and incident CVD may be lower than previously suggested using self-reported data.^43^ This is explained, in part, by differences between self-report and wearables-based measures. Self-reported physical activity is prone to over-reporting due to a combination of social desirability and recall bias,^49 50^ and being limited to measuring blocks of time where an individual may not be active throughout the duration. Wearables provide a continuous objective measure of movement that is not susceptible to the limitations of self-reported physical activity.

### Strengths and limitations

To our knowledge, the current study is among the first aimed to determine the optimal and minimal number of daily steps to lower mortality and incident CVD risk across sedentary time levels. The large sample size and long follow-up allowed us to reduce the risk of reverse causation bias by removing participants with an event in the first 2 years of follow-up, prevalence of major disease, self-rated fair or poor health and who were underweight. Due to the observational design, we cannot rule out the presence of residual and unmeasured confounding. However, E-values indicate for the minimal dose an unmeasured confounder would need to have a moderate association, between 1.46 and 1.81, with the exposures and outcome for the observed relationships to be null. Covariate assessments occurred at a single timepoint and covariates were not treated as time varying.^51^ There was a median lag of 5.5 years between the UK Biobank baseline when covariate measurements were taken and the accelerometry study, although covariates were generally stable over time except for medication.^52 53^ Steps and sedentary time were obtained in a single time point, which can lead to regression dilution bias.^54^ Nevertheless, there was consistent daily steps in participants with repeated measurement 4 years later (n=3400; Kendall’s W=0.74). The UK Biobank had a low response rate and this may contribute to selection bias.^55^ Previous work, however, has shown that the poor representativeness of the UK Biobank sample to the UK population does not materially influence associations with mortality or disease risk.^56^

### Conclusions

In our population-based cohort study of over 70 000 individuals, we did not find an effect modification by sedentary time levels on the dose–response association of daily steps. We found accruing between 9000 and 10 500 steps/day optimally lowered the risk of mortality and incident CVD independent of sedentary time. The minimal threshold associated with substantially lower mortality and CVD risk was between 4000 and 4500 steps/day. We found a lower incident CVD risk for an equivalent number of steps in the low sedentary time group compared with the high sedentary time group. These findings provide tangible targets that can be easily implemented in future steps-based and sedentary time-based interventions, and can inform the first generation of device-based guidelines.

## Data Availability

Data are available on reasonable request. The UK Biobank data that support the findings of this study can be accessed by researchers on application (https://www.ukbiobank.ac.uk/register-apply/).
