# Supplementary material for: Do the associations of daily steps with mortality and incident cardiovascular disease differ by sedentary time levels? A device-based cohort study
Source: Br J Sports Med. 2024 Mar 5;58(5):261–8. doi: 10.1136/bjsports-2023-107221 (PMC10958308; doi:10.1136/bjsports-2023-107221)
Supplement: Supplementary data [file bjsports-2023-107221supp001.pdf]

Supplemental Document

Page	Item
1	Supplemental Figure 1. Flow diagram of participants in the study
2	Supplemental Figure 2: Directed acyclic graph to guide covariate selection
3	Supplemental Figure 3: Joint dose-response association of steps and sedentary time with all-cause mortality
4	Supplemental Figure 4: Joint dose-response association of steps and sedentary time with cardiovascular disease incidence
5	Supplemental Figure 5: Stratified dose-response association of steps, all-cause mortality, and cardiovascular disease incidence by sedentary time. Adjustment for biomarkers
6	Supplemental Figure 6: Stratified dose-response association of steps, all cause mortality, and cardiovascular disease incidence by sedentary time. Exclusion of participants with fair or poor self-rated health, underweight, or had an event within the first two years of follow up
7	Supplemental Figure 7: Stratified dose-response association of all-cause mortality and steps by sedentary time; highest quartile and lowest three quartile grouping.
8	Supplemental Figure 8: Stratified dose-response association of cardiovascular disease incidence and steps by sedentary time; highest quartile and lowest three quartile grouping
9	Supplemental Figure 9: Stratified dose-response association of cardiovascular disease incidence and steps by sedentary time; cause-specific analysis
10	Supplemental Figure 10: Stratified dose-response association of cardiovascular disease incidence and steps by sedentary time; age subgroups.

11	**Supplemental Table 1: Assessment of CVD and cancer incidence* and definition of diseases**
12	**Supplemental Table 2: Covariate definitions**
13	**Supplemental Table 3: Crude absolute risk stratified by steps and sedentary behaviour groups**
14	**Supplemental Table 4: E-values for optimal and minimal steps/day for all-cause mortality and cardiovascular disease incidence by sedentary time**
15	**Supplemental Text 1: Physical activity, sedentary behaviour, and step classification**

Supplemental Figure 1. Flow diagram of participants in the study

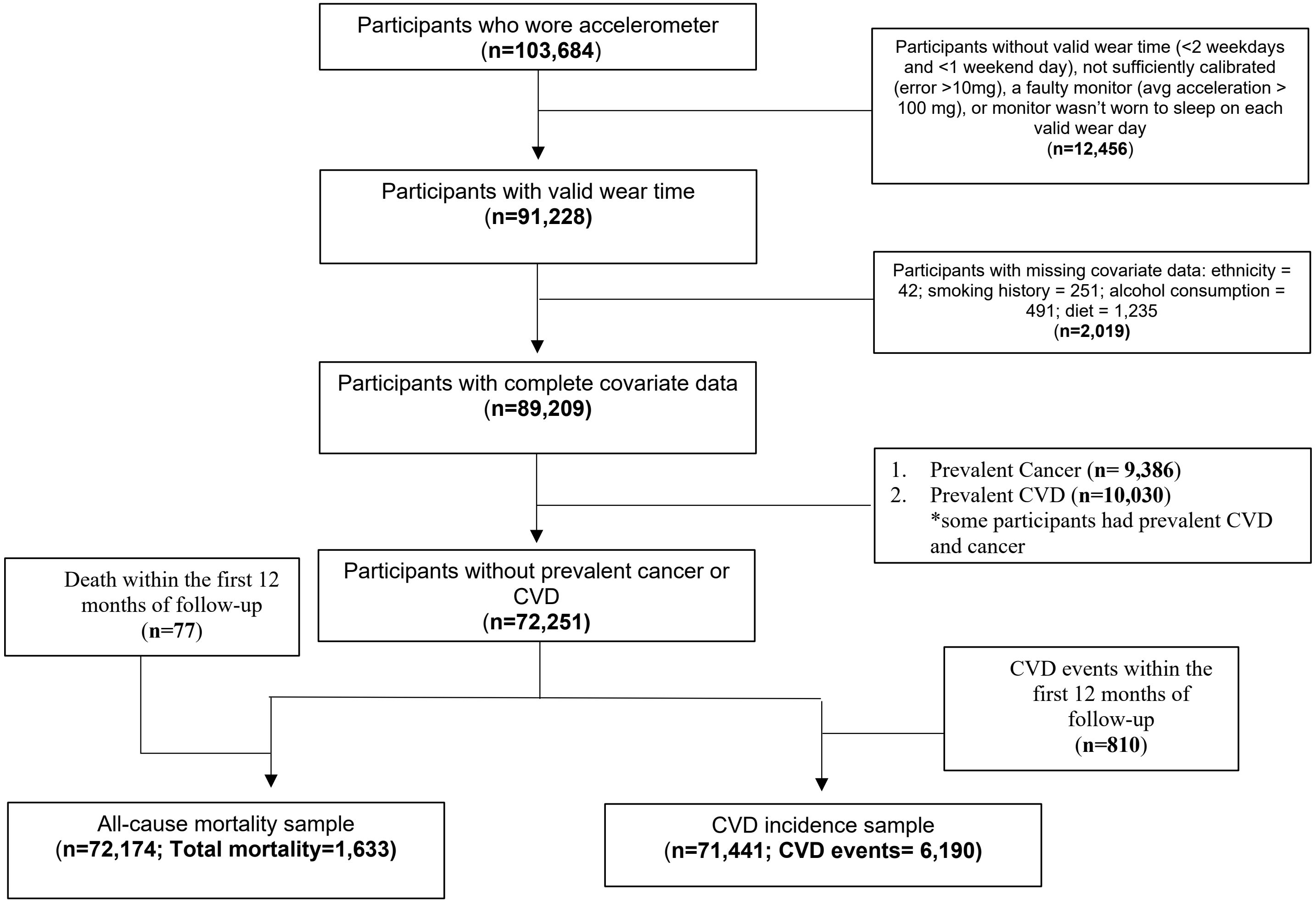

1

Supplemental Figure 2: Directed acyclic graph to guide covariate selection

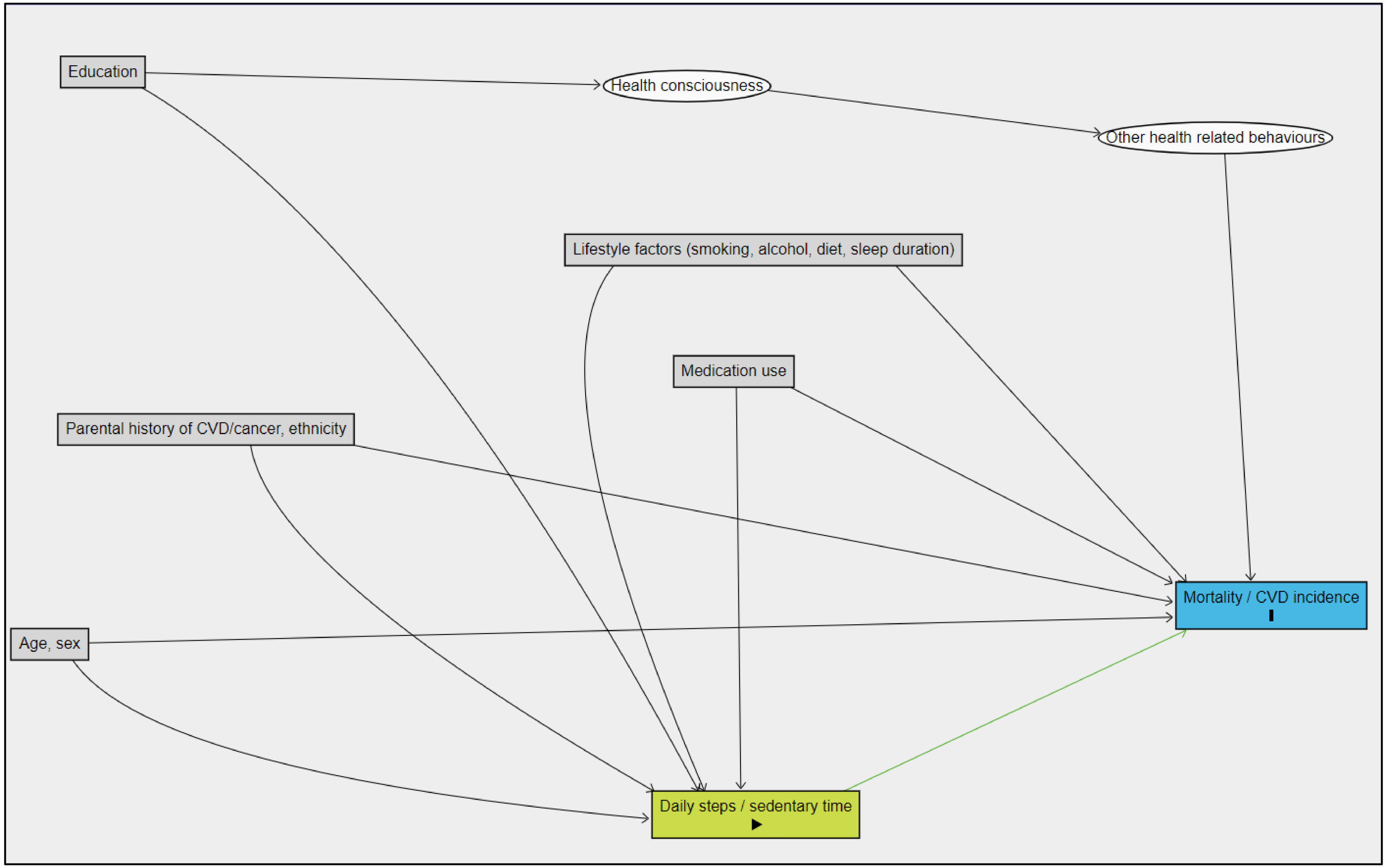

Green= exposure; blue= outcome; grey= adjusted variables; white= unobserved variables

Supplemental Figure 3: Joint dose-response association of steps and sedentary time with all-cause mortality

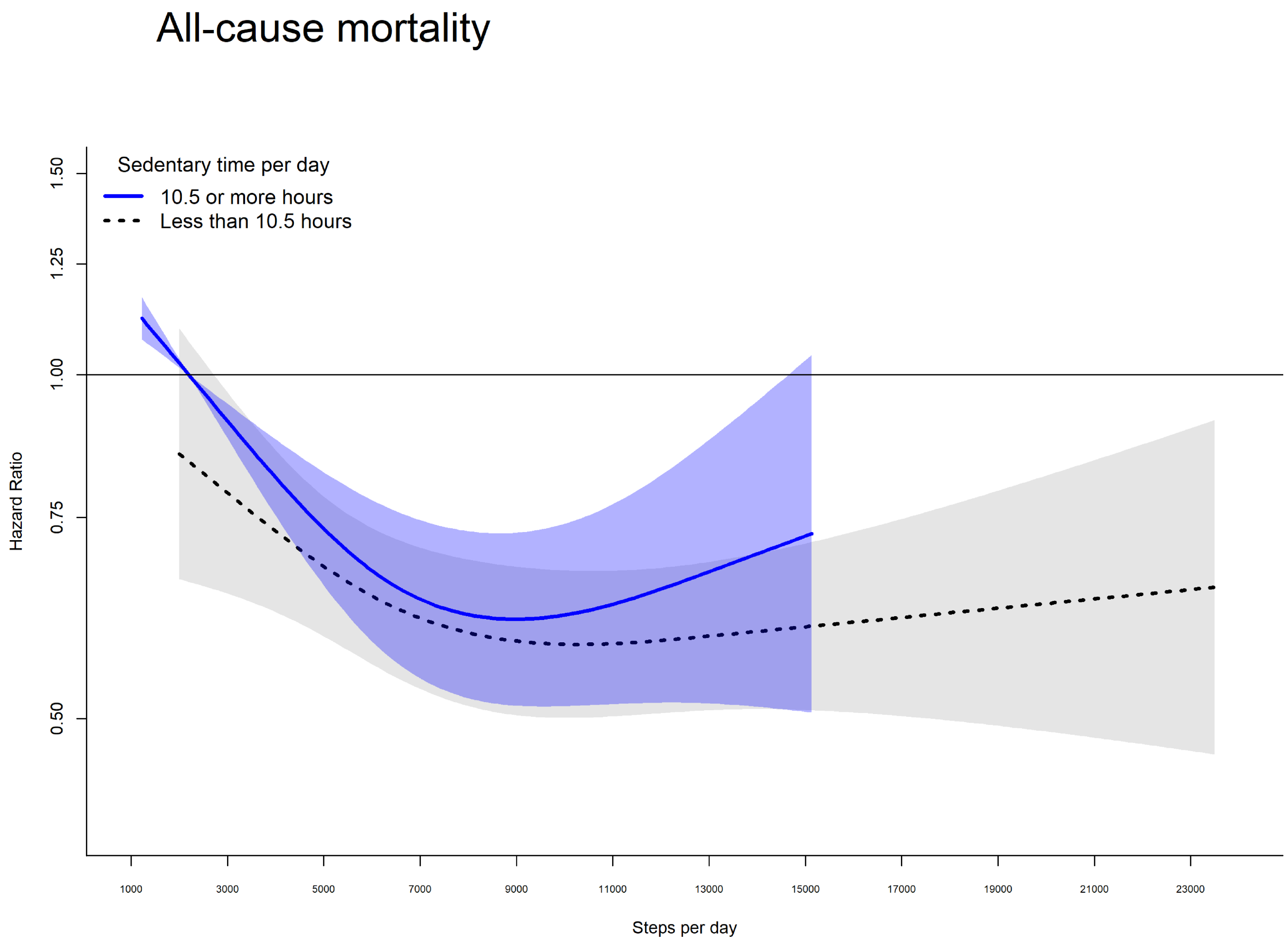

Adjusted for age, sex, ethnicity, education, smoking status, alcohol consumption, diet, parental history of CVD and cancer, medication use (cholesterol, insulin, and hypertension), sleep duration. Shaded area represents 95%CI

3

Supplemental Figure 4: Joint dose-response association of steps and sedentary time with cardiovascular disease incidence

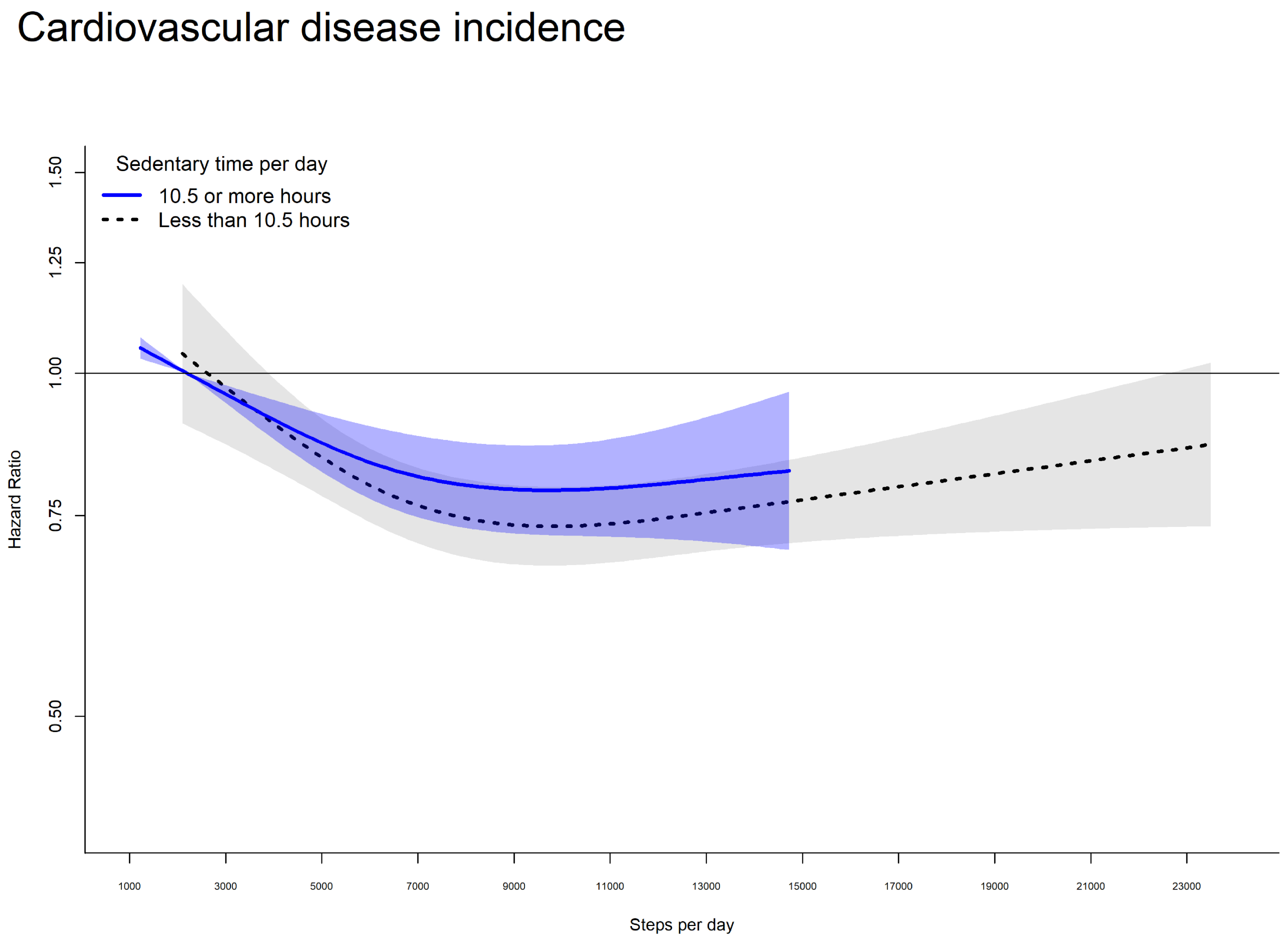

Adjusted for age, sex, ethnicity, education, smoking status, alcohol consumption, diet, parental history of CVD and cancer, medication use (cholesterol, insulin, and hypertension), sleep duration. Shaded area represents 95%CI

4

Supplemental Figure 5: Stratified dose-response association of steps, all-cause mortality, and cardiovascular disease incidence by sedentary time. Adjustment for biomarkers

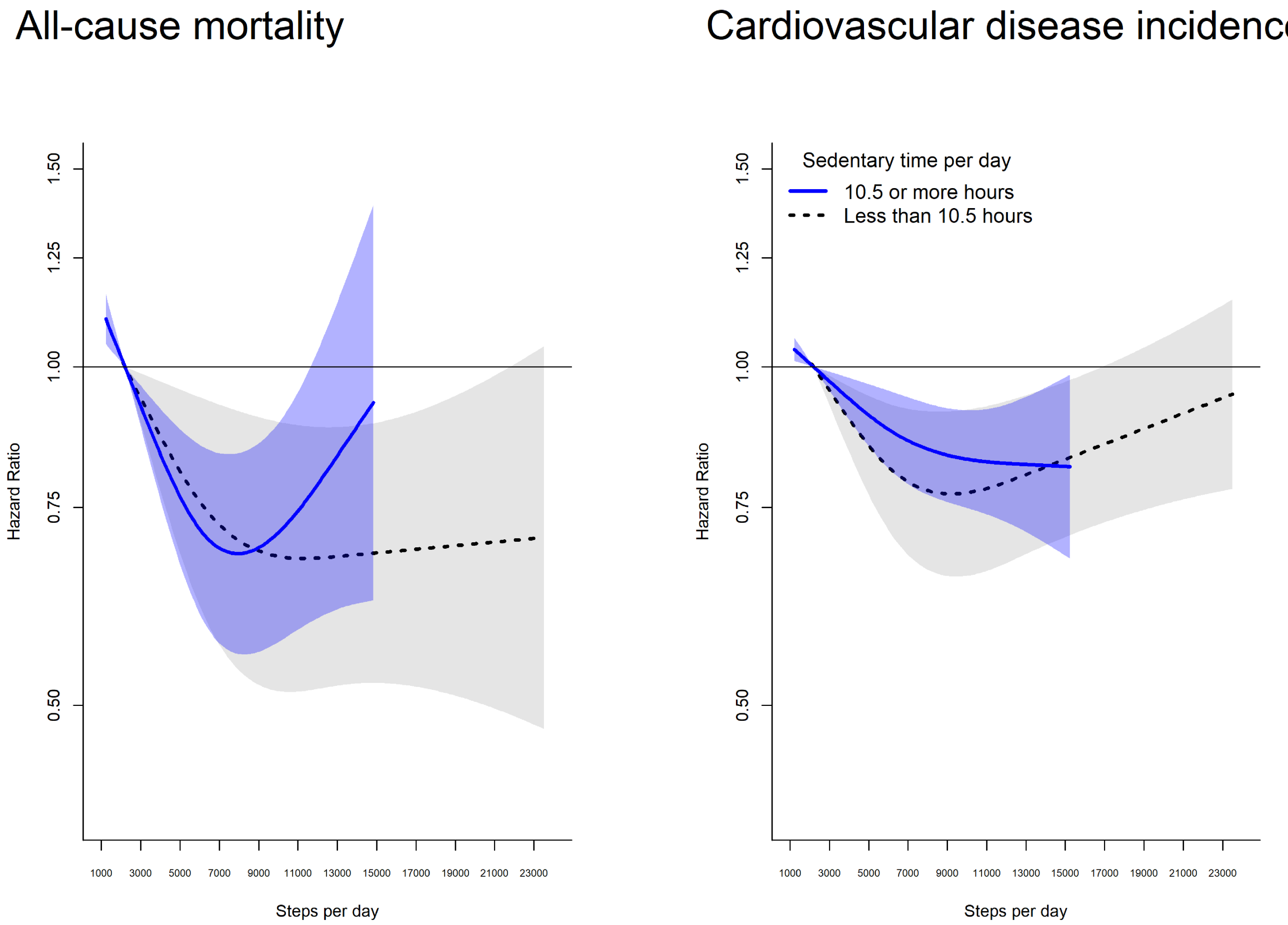

Adjusted for age, sex, ethnicity, education, smoking status, alcohol consumption, diet, parental history of CVD and cancer, medication use (cholesterol, insulin, and hypertension), sleep duration, waist circumference, glycated hemoglobin A1C, high-density and low-density lipoprotein, blood pressure, and triglycerides. Shaded area represents 95%CI

Supplemental Figure 6: Stratified dose-response association of steps, all cause mortality, and cardiovascular disease incidence by sedentary time. Exclusion of participants with fair or poor self-rated health, underweight, or had an event within the first two years of follow up

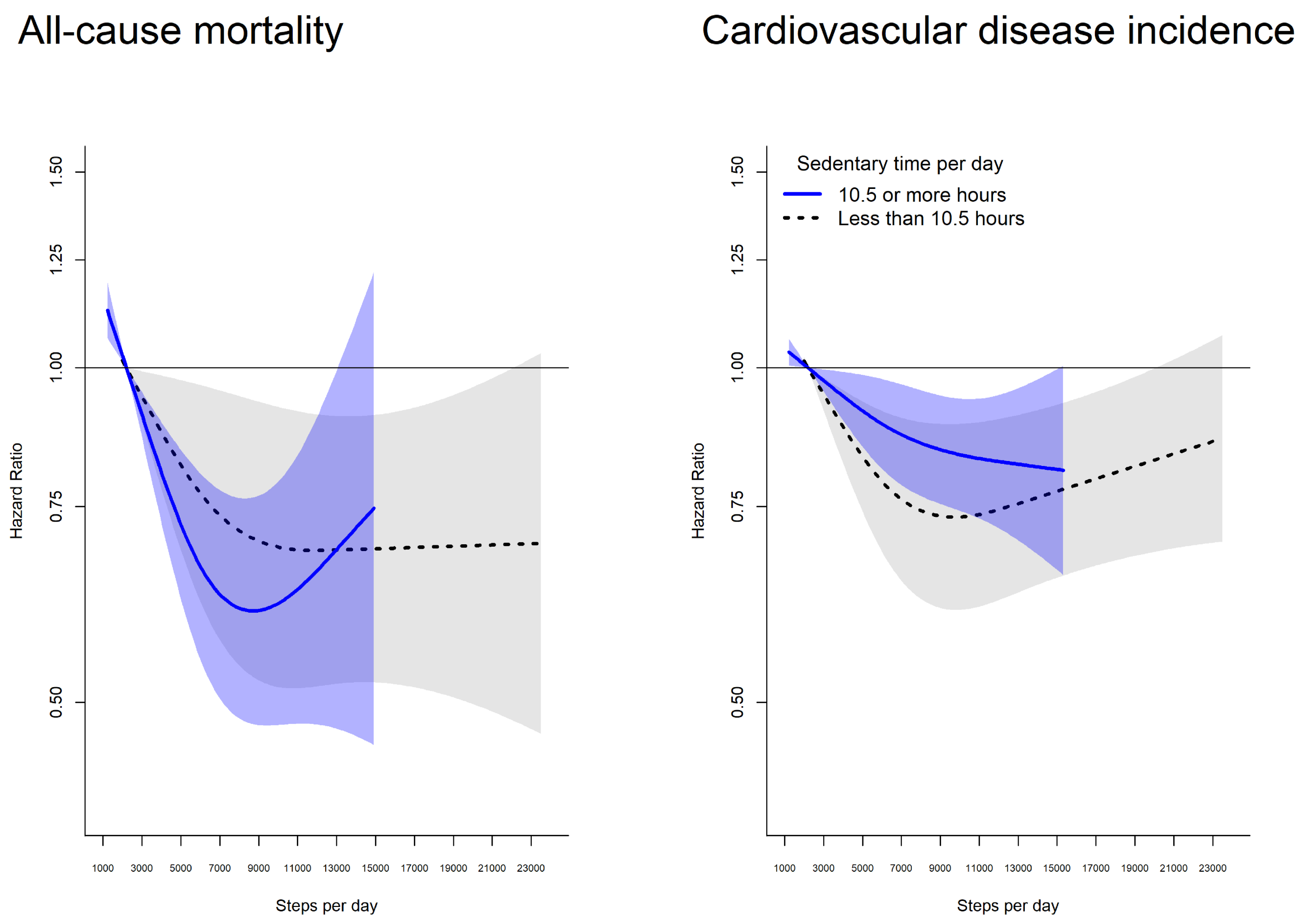

Adjusted for age, sex, ethnicity, education, smoking status, alcohol consumption, diet, parental history of CVD and cancer, medication use (cholesterol, insulin, and hypertension) and sleep duration. Shaded area represents 95%CI

6

Supplemental Figure 7: Stratified dose-response association of all-cause mortality and steps by sedentary time; highest quartile and lowest three quartile grouping.

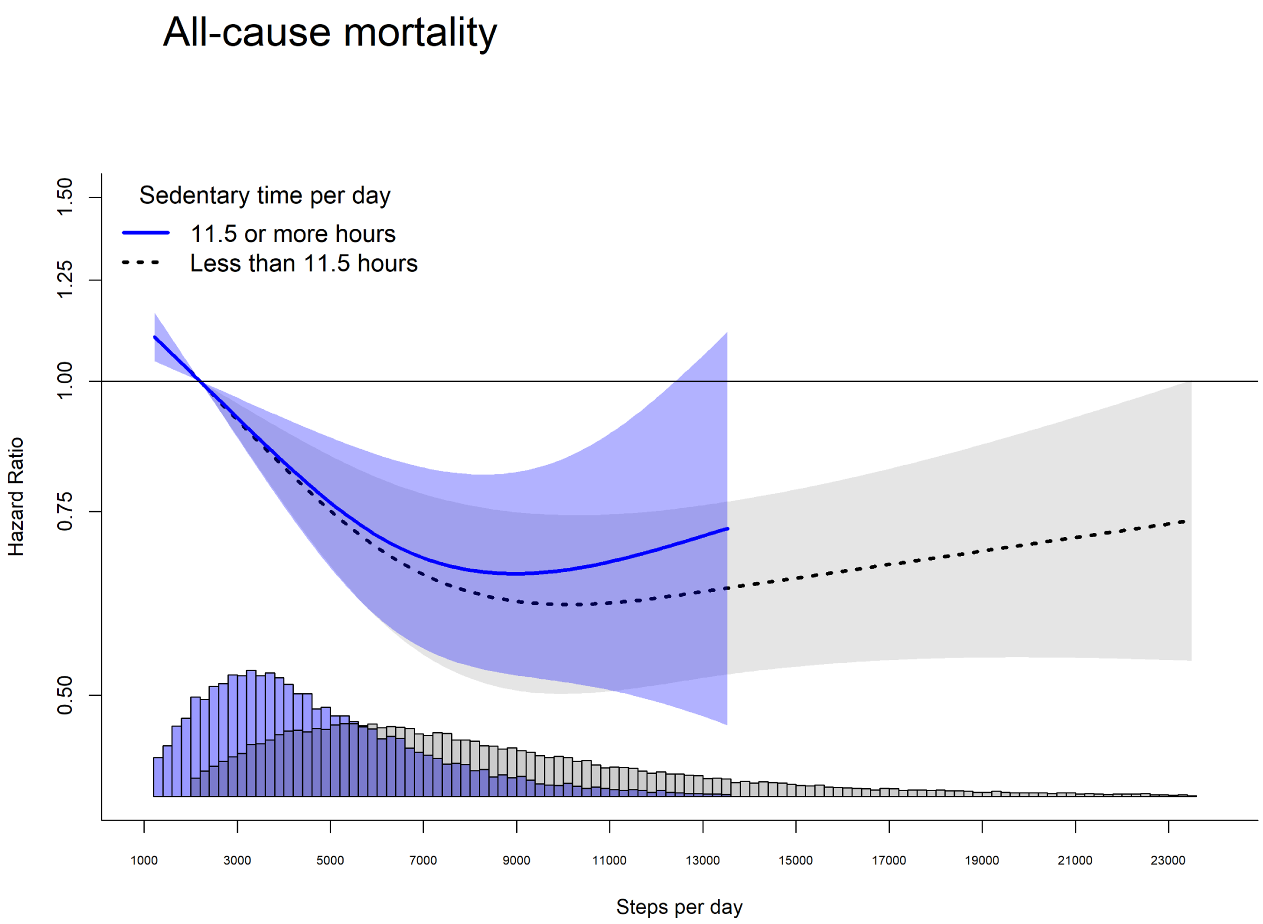

Adjusted for age, sex, ethnicity, education, smoking status, alcohol consumption, diet, parental history of CVD and cancer, medication use (cholesterol, insulin, and hypertension) and sleep duration. Shaded area represents 95%CI

7

Supplemental Figure 8: Stratified dose-response association of cardiovascular disease incidence and steps by sedentary time; highest quartile and lowest three quartile grouping.

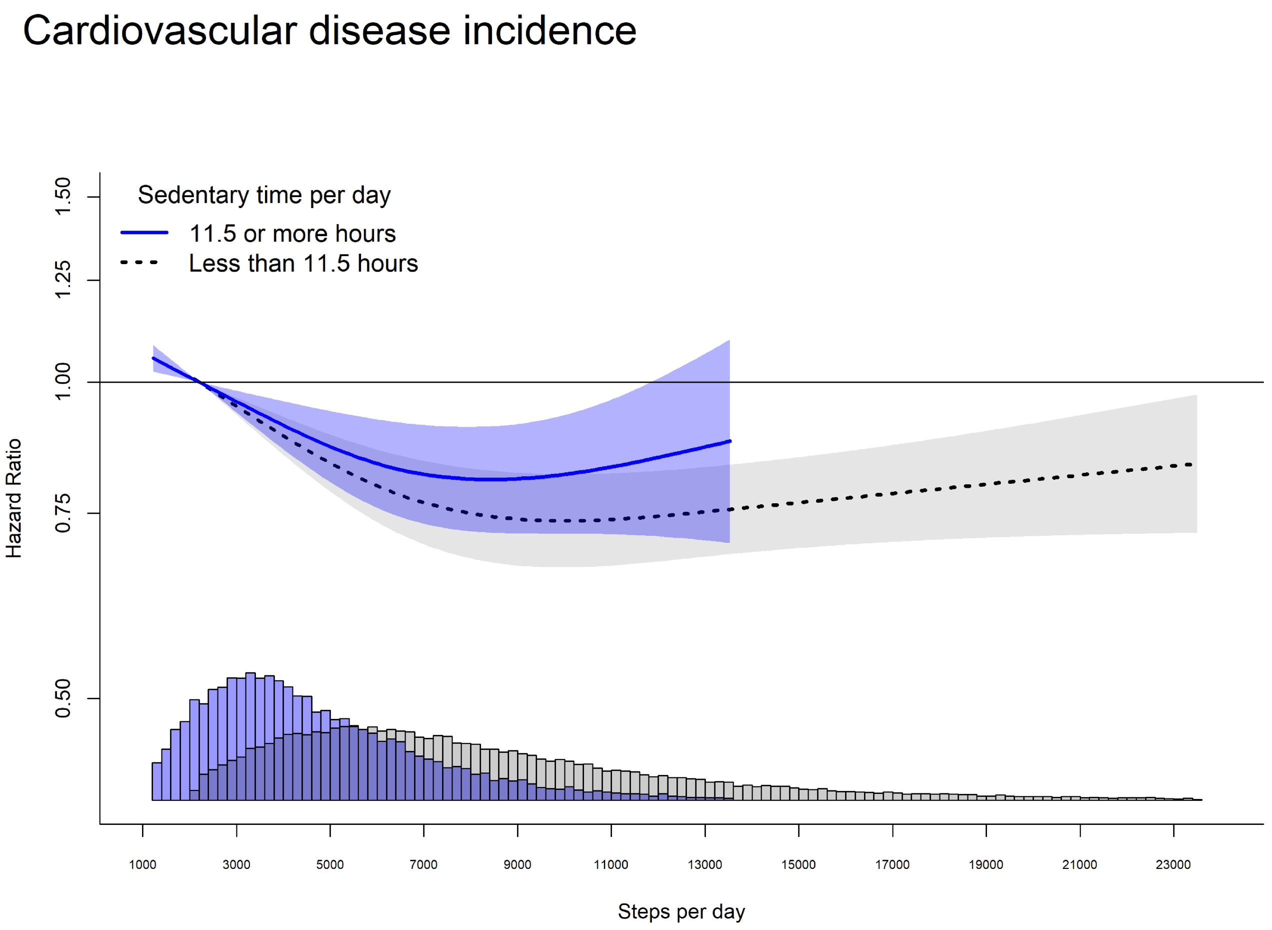

Adjusted for age, sex, ethnicity, education, smoking status, alcohol consumption, diet, parental history of CVD and cancer, medication use (cholesterol, insulin, and hypertension) and sleep duration. Shaded area represents 95%CI

Supplemental Figure 9: Stratified dose-response association of cardiovascular disease incidence and steps by sedentary time; cause-specific analysis.

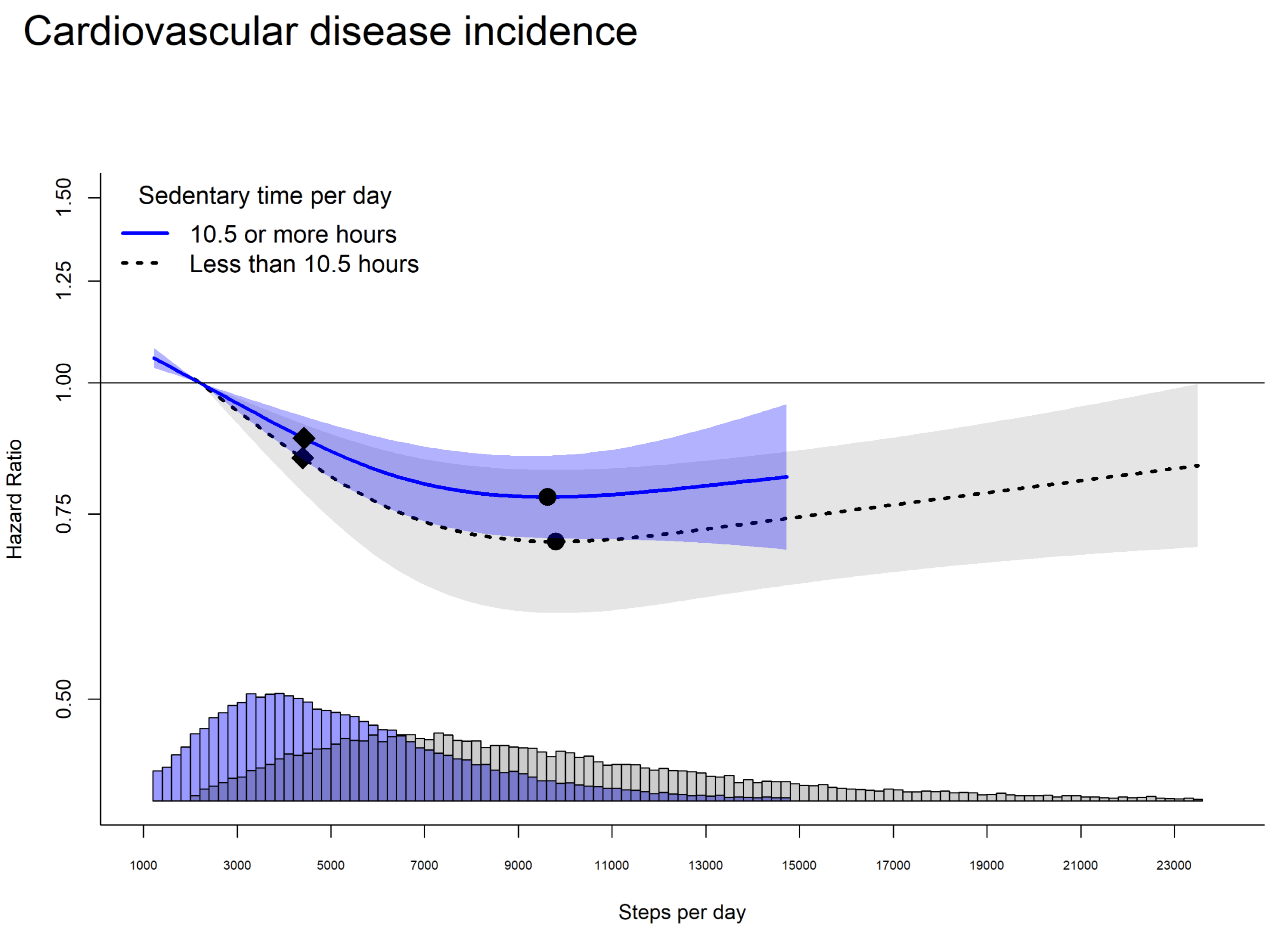

Adjusted for age, sex, ethnicity, education, smoking status, alcohol consumption, diet, parental history of CVD and cancer, medication use (cholesterol, insulin, and hypertension) and sleep duration. Shaded area represents 95%CI. Square= minimum dose (ED50); circle= optimum dose (nadir of curve)

BMJ Publishing Group Limited (BMJ) disclaims all liability and responsibility arising from any reliance placed on this supplemental material which has been supplied by the author(s)

Supplemental Figure 10: Stratified dose-response association of cardiovascular disease incidence and steps by sedentary time; age subgroups.

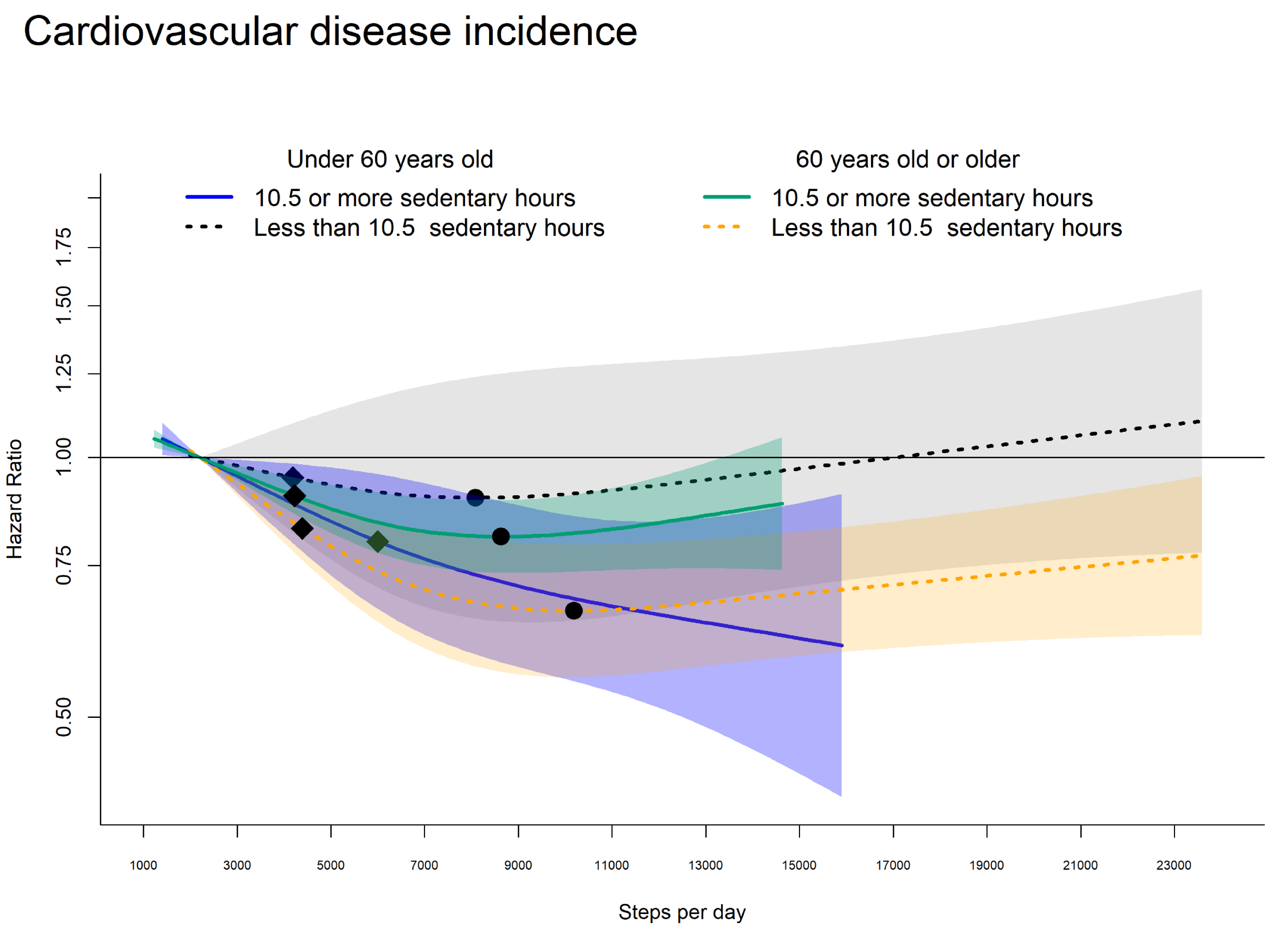

Adjusted for sex, ethnicity, education, smoking status, alcohol consumption, diet, parental history of CVD and cancer, medication use (cholesterol, insulin, and hypertension) and sleep duration. Shaded area represents 95%CI. Square= minimum dose (ED50); circle= optimum dose (nadir of curve)

10

Supplemental Table 1: Assessment of CVD and cancer incidence* and definition of diseases

Variable	Definition
Inpatient hospitalisation	The inpatient hospitalization data were provided by either the Hospital Episode Statistics for England, the Patient Episode Database for Wales, or the Scottish Morbidity Record for Scotland
Cardiovascular disease definition	CVD was defined as diseases of the circulatory system, excluding hypertension, diseases of arteries, and lymph. The ICD-10 codes included were: I0, I11, I13, I20-I51, I60-I69.

*Incident events included fatal and nonfatal events

Supplemental Table 2: Covariate definitions

Variable	Definition	UK Biobank field ID (if applicable)
Age	Continuous (years)	34, 52, accelerometer date-timestamp
Sex	Female/Male	31
Ethnicity	Asian, Black, Mixed, Other, White	21000
Smoking status	Never, past, current	20116
Alcohol consumption	Units/week; 1 unit = 8g of pure ethanol	20117, 1558
Sleep duration	Hours spent sleeping	Derived from accelerometer data
Diet	Fruits and vegetables servings/day	1309, 1319, 1289, 1299
Education	College/University; A/AS level; O levels; CSE; NVQ/HND/HNC; other	6138
Parental history of CVD	Self-reported mother or father diagnosed with heart disease or stroke	20107, 20110
Parental history of cancer	Self-reported mother or father diagnosed with prostate cancer, breast cancer, bowel cancer, or lung cancer	20107, 20110
Use of cholesterol medication	Yes/No	6177, 6153
Use of blood pressure medication	Yes/No	6177, 6153
Use of diabetes medication	Yes/No	6177, 6153
Glycated hemoglobin A1C	Continuous; mmol/mol	30750
High density lipoprotein	Continuous; mmol/mol	30760
Low density lipoprotein	Continuous; mmol/mol	30780
Diastolic blood pressure	Continuous; mmHg	4079
Systolic blood pressure	Continuous; mmHg	4080
Triglycerides	Continuous; mmol/L	30870
Waist Circumference	Continuous; cm	48

Ahmadi MN, *et al. Br J Sports Med* 2024; 58:261–268. doi: 10.1136/bjsports-2023-107221

Supplemental Table 3: Crude absolute risk stratified by steps and sedentary behaviour groups

Steps and sedentary time	Total Mortality	Cardiovascular disease incidence
**High ST**		
<4,000 steps	5.41 (5.32, 5.50)	19.85 (19.53, 20.18)
4,000 to 8,000 steps	3.40 (3.34, 3.45)	15.58 (15.35, 15.80)
>8,000 steps	3.05 (2.96, 3.13)	13.51 (13.18, 13.84)
**Low ST**		
<4,000 steps	3.74 (3.62, 3.86)	16.54 (15.93, 17.15)
4,000 to 8,000 steps	2.43 (2.39, 2.47)	11.70 (11.50, 11.90)
>8,000 steps	2.27 (2.24, 2.30)	10.95 (10.79, 11.11)

Values represent percent and (95% CI's); High ST ≥10.5 hours/day; Low ST <10.5 hours/day; stepping categories are based on tertiles

Supplemental Table 4: **E-values for optimal and minimal steps/day for all-cause mortality and cardiovascular disease incidence by sedentary time**

	Steps/day	Total Mortality
**High ST**		
Minimal dose	4,100	1.81 (1.56)
Optimal dose	9,000	2.66 (2.08)
**Low ST**		
Minimal dose	4,400	1.67 (1.21)
Optimal dose	10,300	2.26 (1.39)

	Steps/day	Cardiovascular disease incidence
**High ST**		
Minimal dose	4,300	1.46 (1.32)
Optimal dose	9,700	1.85 (1.60)
**Low ST**		
Minimal dose	4,300	1.60 (1.39)
Optimal dose	9,800	2.17 (1.70)

Supplemental Text 1: Physical activity, sedentary behaviour, and step classification

Physical activity was classified using a previously validated Random Forest (RF) activity classifier. RF is an ensemble of multiple decision trees. Each tree is learned on a bootstrap sample of training data and each node in the tree is split using the best among a randomly selected set of acceleration features. The decisions from each tree are aggregated and a final model prediction is based on majority vote. The RF model requires very little pre-processing of the data, as the features do not need to be normalized. Additionally, the model is resistant to over fitting the training data because each tree within the forest is independently grown to maximum depth using a randomly selected subset of features.

The classifier categorized physical activity in 10 second windows into 1 of 4 activity classes: sedentary, standing utilitarian movements (ironing a shirt, washing dishes), walking activities (gardening, active commuting, mopping floors), running/high energetic activities (active playing with children). For the 2-level step detection, these activities were further assigned as non-ambulatory and ambulatory activities. The diagram below depicts how activity was classified at both activity levels. For ambulatory activities, a previously validated signal peak detection step count algorithm was applied. The algorithm detects peaks in the acceleration signal and applies a series of parameters (peak magnitude, periodicity, and continuity) to identify peaks that represent steps. These peaks are then summed within each ambulatory window.

Activity, sedentary behaviour, and stepping differentiation from sleep and non-wear was identified using the change in tilt angle and acceleration standard deviation. Monitors were calibrated and corrected for orientation using previously published methods.

Physical activity type and ambulatory classification

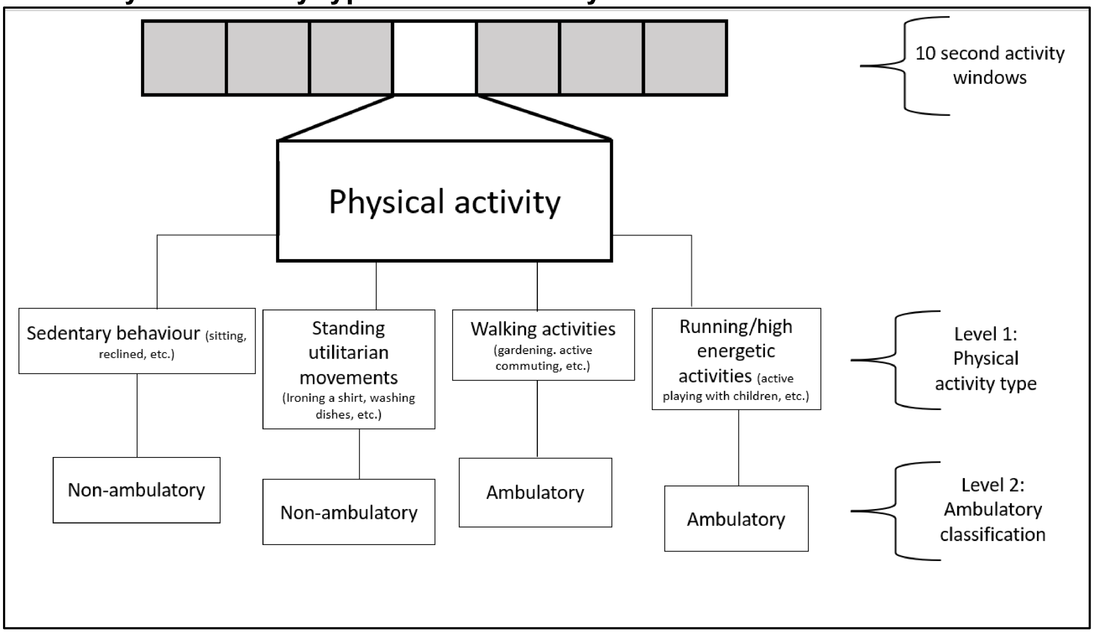

Although previously validated, to assess the robustness and generalizability of the sedentary behaviour and ambulatory classification, performance was evaluated in an independent sample of 211 participants (Age range = 18 to 91; 60.6% female) performing structured and free-living activities from the US (University of California Irvine Center for Machine Learning and Intelligent Systems *Physical Activity Monitoring for Aging People* study [published data], accessible at https://archive.ics.uci.edu/ml/datasets and Clemson University *Shimmer3 Pedometer Dataset*

[published data], accessible at https://sites.google.com/view/rmattfeld/pedometer-dataset?authuser=0), Australia (University of Sydney *Intermittent Lifestyle Physical Activity* Study [unpublished data]) and UK (University of Oxford *Capture 24* study [published data], accessible at https://ora.ox.ac.uk/objects/uuid:99d7c092-d865-4a19-b096-cc16440cd001). Because of the picture-based ground truth provided in the *Capture 24* dataset, a ground-truth label had to be consistent for at least 5 minutes to be extracted. A total of 139,944 activity samples (23,324 minutes) were collected. For free-living activities participant-worn body-cameras, or researcher-held Go-Pro video-recordings were used to attain ground-truth physical activity. The activity coding procedures have been previously described. Classification performance was evaluated using overall accuracy, kappa statistic, recall, precision, and F1-score.

Step detection was evaluated in 60 of the participants who had ground-truth step counts using video direct observation or a thigh-worn monitor that has a 99% accuracy with directly observed steps. Step detection performance was evaluated using Pearson Correlation, intraclass correlation coefficient (two-way fixed mixed effects model), mean absolute percent error, and mean bias. To assess the accuracy under a variety of walking conditions, in a subsample of 30 participants with available data, we further evaluated performance by separating different walking speeds (treadmill-based), walking under free-living conditions, and walking very fast/running under free-living conditions.

Activity classification performance in the four datasets:

	Sensitivity	Precision	F1-score	Kappa statistic
Ambulation	86.2 (82.2, 90.2)	96.0 (93.4, 98.6)	90.8 (86.3, 95.3)	0.84 (0.79, 0.89)
Sedentary	91.5 (86.2, 96.8)	72.5 (66.1, 78.9)	80.9 (73.1, 88.7)	0.72 (0.66, 0.78)

Step detection in the 60 participants with ground-truth step data:

	Correlation (95% CI)	Intraclass correlation coefficent (95% CI)	Mean absolute percent error (SD)	Steps mean bias (SD)
Steps	0.96 (0.93, 0.98)	0.86 (0.77, 0.92)	10.6% (9.2%)	103 (±152)

Ground-truth step total: mean (SD)= 1,254 (±545) median [IQR]= 1,219 [907, 1,658]

Ahmadi MN, *et al. Br J Sports Med* 2024; 58:261–268. doi: 10.1136/bjsports-2023-107221

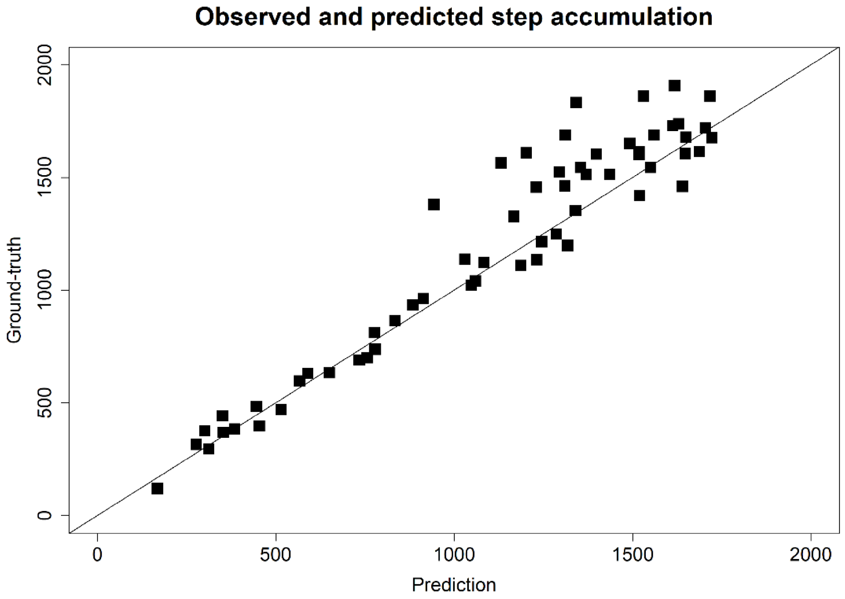

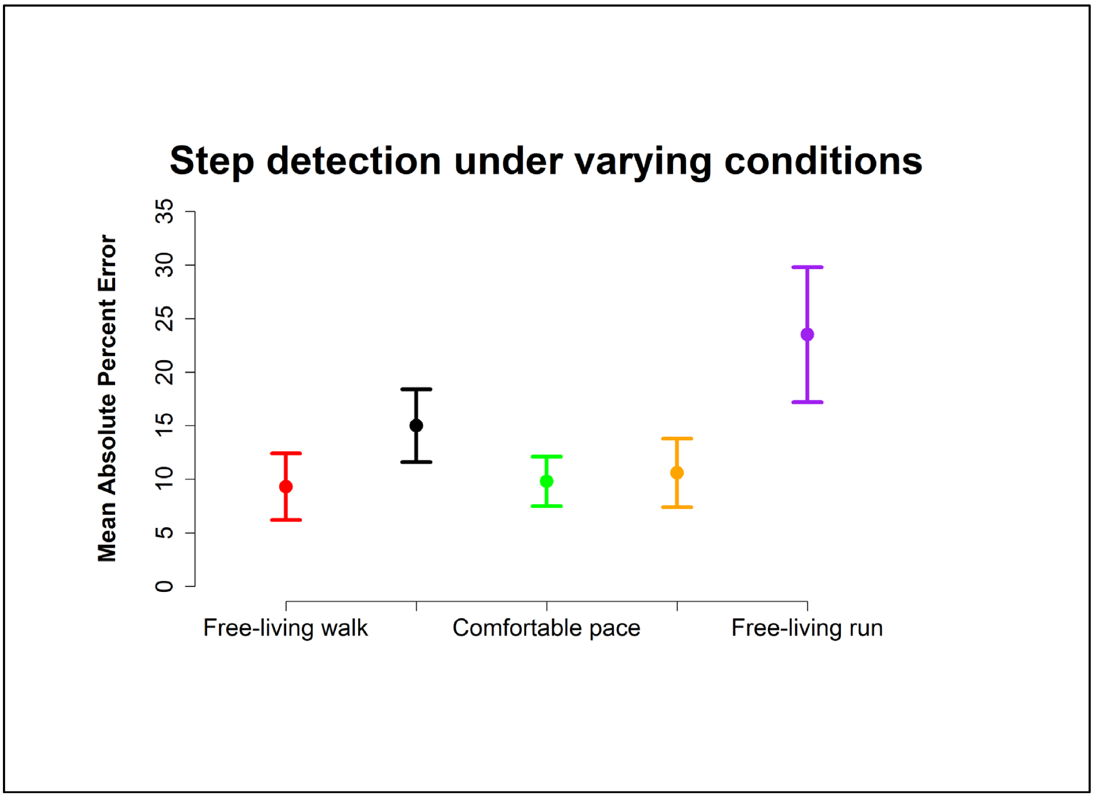

Slow, comfortable, and fast paced walking were based on treadmill speeds and participant preferences that corresponded to <3 km/h, 3-5 km/h, and >6 km/h, respectively. Error bars = 95% CI
